# Complete Genome Sequence of Pronghorn Virus, a Pestivirus

**DOI:** 10.1128/genomeA.00575-14

**Published:** 2014-06-12

**Authors:** John D. Neill, Julia F. Ridpath, Nicole Fischer, Adam Grundhoff, Alexander Postel, Paul Becher

**Affiliations:** a U.S. Department of Agriculture, Agricultural Research Service, National Animal Disease Center, Ruminant Diseases and Immunology Research Unit, Ames, Iowa, USA; b Institute for Medical Microbiology, Virology, and Hygiene, University Medical Center Hamburg-Eppendorf, Hamburg, Germany; c Heinrich Pette Institute, Leibniz Institute for Experimental Virology, Research Group Virus Genomics, Hamburg, Germany; d Institute of Virology, University of Veterinary Medicine, Hannover, Germany; e German Center for Infection Research (DZIF), Partner Site Hamburg-Lübeck-Borstel, Hamburg, Germany; f German Center for Infection Research (DZIF), Partner Site Hannover-Braunschweig, Hannover, Germany

## Abstract

The complete genome sequence of pronghorn virus, a member of the *Pestivirus* genus of the family *Flaviviridae*, was determined here. The virus, originally isolated from a pronghorn antelope, has a genome of 12,273 nucleotides, with a single open reading frame of 11,694 bases encoding 3,897 amino acids.

## GENOME ANNOUNCEMENT

The *Pestivirus* genus of the family *Flaviviridae* is composed of a group of agriculturally important viruses. The recognized species of the genus include bovine viral diarrhea virus 1 (BVDV-1), BVDV-2, classical swine fever virus, and border disease virus (1). Putative species include Bungowannah virus, giraffe virus, HoBi-like virus, and pronghorn virus. These viruses have a single-stranded, positive-sense, nonpolyadenylated genomic RNA that contains a single large open reading frame (ORF) encoding all viral proteins. The first and only isolation of pronghorn virus to date was from an immature blind pronghorn antelope at the Wyoming State Veterinary Laboratory (Cheyenne, WY). Preliminary characterization of the virus has been reported (2). Sequencing analysis revealed that pronghorn virus was a pestivirus, having the N-terminal protease (N^pro^), the hallmark of the pestiviruses. The pronghorn isolate belongs to the noncytopathic biotype and grows in both ovine- and bovine-derived cell lines. Pronghorn virus was considered the most divergent pestivirus until the isolation and sequencing of Bungowannah virus (3). Because this is the only example of this virus isolated to date, little is known concerning its host range, pathogenic potential, or prevalence.

Sequencing of the genomic RNA of pronghorn virus from the cell culture supernatant was done using the Ion Torrent (4, 5) and Illumina MiSeq (6, 7) sequencing protocols, as previously described. The 3′ end of the genomic RNA was confirmed by 3′ RACE using a primer set designed to amplify the 3′ terminal 600 bases.

Sequencing of the pronghorn virus library by Ion Torrent yielded 84,025 (63,403 virus) individual sequences, with an average depth of coverage of 712.6×. Illumina sequencing provided 4,340,978 reads with 700,290 pestivirus sequence reads. The pestiviral sequences were assembled in one single contig, with a median coverage of 4,290.5×. Following assembly of the complete genomic RNA using data from both platforms, the genome length was determined to be 12,273 bases, with a single ORF of 11,694 bases (3,897 amino acids). The 5′ and 3′ untranslated regions (UTR) comprise 369 and 210 nucleotides, respectively. These are similar in length to those reported for other pestiviruses. There are seven nucleotide differences between the sequences determined by the two methods (T2289A, A2631G, A3536C, G5720A, G8364T, G8574A, and C10465T) that most likely resulted from subculturing of the virus. In addition, two nucleotide residues within the 3′ UTR (A12257 and T12260) were absent in the genomic sequence determined by Illumina sequencing, which most likely resulted from subculturing of the virus. In the earlier report describing pronghorn virus, the nucleotide sequences of N^pro^ and the structural proteins were described. The amino acid alignments of the N^pro^ and E2 proteins demonstrated a significant genetic distance from other pestiviruses. Similar analyses of the pronghorn virus nonstructural proteins and complete genome sequences further support the conclusion that this virus should be considered a separate species among the pestiviruses.

### Nucleotide sequence accession number.

The complete genomic sequence of pronghorn virus has been deposited in GenBank under the accession no. AY781152.
